# The mol­ecular and crystal structures of 2-(3-hy­droxy­prop­yl)benzimidazole and its nitrate salt

**DOI:** 10.1107/S2056989022000585

**Published:** 2022-01-20

**Authors:** Dilnoza Rakhmonova, Zukhra Kadirova, Batirbay Torambetov, Shakhnoza Kadirova, Jamshid Ashurov, Svitlana Shishkina

**Affiliations:** a National University of Uzbekistan named after Mirzo Ulugbek, 4 University St, Tashkent, 100174, Uzbekistan; b Institute of Bioorganic Chemistry, Academy of Sciences of Uzbekistan, M. Ulugbek Str, 83, Tashkent, 700125, Uzbekistan; c State Scientific Institution "Institute for Single Crystals" of National Academy of Sciences of Ukraine, 60 Nauky ave., 61001 Kharkiv, Ukraine

**Keywords:** mol­ecular structure, crystal structure, 2-(3-hy­droxy­prop­yl)benzimidazole, hydrogen bond, Hirshfeld analysis, periodic calculations

## Abstract

2-(3-Hy­droxy­prop­yl)-1*H*-benzimidazole, C_10_H_12_N_2_O, which has potential biological activity, can be used as a ligand for complexation with metals. This compound is an electron donor, due to the lone pair of the nitro­gen atom in the imidazole ring. This nitro­gen atom also acts as a proton acceptor. In the crystalline phase, the nitrate salt, namely, 2-(3-hy­droxy­prop­yl)-1*H*-benzim­id­azol-3-ium nitrate, C_10_H_13_N_2_O^+^·NO_3_^−^, has been studied. The protonation of the 2-(3-hy­droxy­prop­yl)benzimidazole unit results in significant delocalization of the electron density within the imidazole ring. The salt formation leads to variations in the inter­molecular inter­actions, which were studied by analysis of the Hirshfeld surfaces and two-dimensional fingerprint plots.

## Abstract 2

The mol­ecular and crystal structures of 2-(3-hy­droxy­prop­yl)-1*H*-benzimidazole and of its nitrate salt have been studied. Hirshfeld surfaces and fingerprint plots were generated to investigate the inter­molecular inter­actions.

## Chemical context

Benzimidazole derivatives and their complex compounds possess a wide spectrum of biological activity (Salahuddin *et al.*, 2012 ▸), including anti­bacterial (Chkirate *et al.*, 2020 ▸), anti­fungal (Khabnadideh *et al.*, 2012 ▸), anti­viral (Kharitonova *et al.*, 2017 ▸), anti­parasitic (Katti *et al.*, 2019 ▸), anti-inflammatory and analgesic (Gaba *et al.*, 2014 ▸) activities.

Nitro­gen-containing heterocycles can be lone-pair donors, forming complex compounds with a metal; in some, the nitro­gen heterocycle binds to the metal atom (Mottillo *et al.*, 2015 ▸). The lone pair of the cyclic nitro­gen atom can be protonated, forming an organic cation (Yan *et al.*, 2009 ▸; Yu *et al.*, 2007 ▸, Bayar *et al.*, 2018 ▸; Chen *et al.*, 2010 ▸). It has been shown (Pilipenko & Tananaiko, 1983 ▸) that compounds containing a protonated cation are formed as a result of the combination with counter-ions. Such compounds, also called ionic associates, are inter­mediate compounds between simple salts and complex (coordination) compounds. They have properties similar to those of mixed-ligand complexes, although the properties of the compound as a whole depends on many factors. 
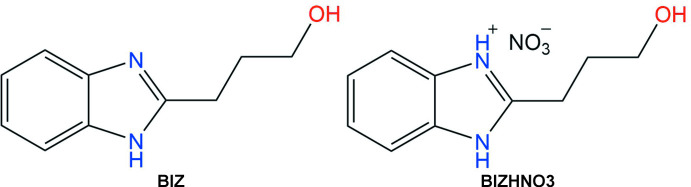


In the present paper we report the mol­ecular and crystal structures of 2-(3-hy­droxy­prop­yl)benzimidazole (BIZ) and its nitrate salt (BIZHNO3), which were determined to study the influence of protonation.

## Structural commentary

**Figure 1:**
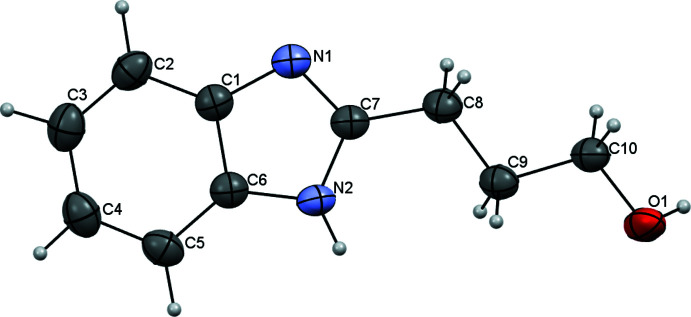
Mol­ecular structure of the neutral 2-(3-hy­droxy­prop­yl)benzimidazole mol­ecule in the BIZ structure. Displacement ellipsoids are shown at the 50% probability level.

**Figure 2:**
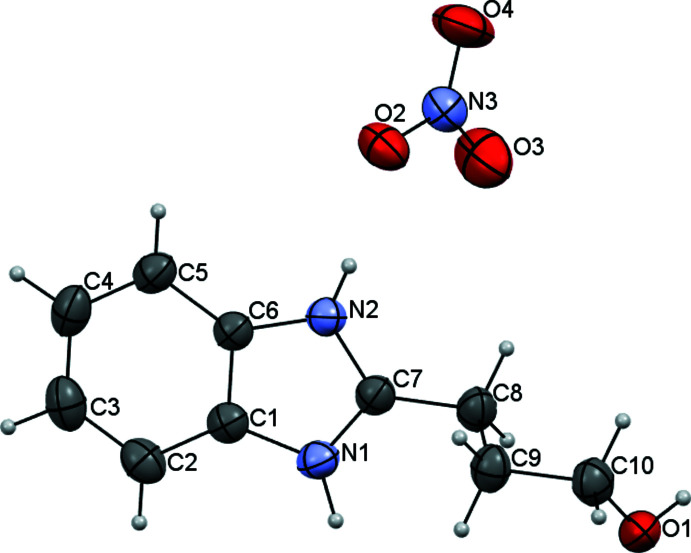
Mol­ecular structure of the 2-(3-hy­droxy­prop­yl)benzimidazole nitrate salt in the BIZHNO3 structure. Displacement ellipsoids are shown at the 50% probability level.

Analysis of the mol­ecular structures of the title compounds revealed that the C7—N1 and C7—N2 bonds have different lengths [N1—C7 = 1.322 (4) Å and N2—C7 = 1.352 (4) Å] in the neutral BIZ mol­ecule (Fig. 1 ▸) but are equal within standard uncertainties [N1—C7 = 1.329 (2) Å and N2—C7 = 1.331 (2) Å] in its protonated form in BIZHNO3 (Fig. 2 ▸). Such a delocalization of the electron density during protonation allows the structure of protonated BIZ mol­ecule to be described as a superposition of two resonance structures, as shown in the scheme below. 
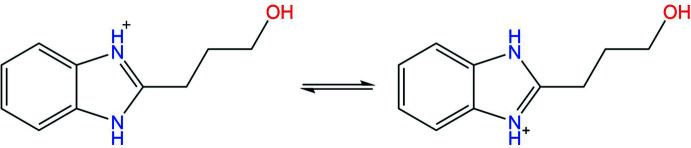


The neutral and protonated BIZ mol­ecules differ in the conformation of the hy­droxy­alkyl substituent (Figs. 1 ▸ and 2 ▸). In the neutral BIZ mol­ecule, the hy­droxy­alkyl substituent is almost coplanar to the benzimidazole fragment [the N2—C7—C8—C9 torsion angle is 15.3 (4)°]. The hy­droxy­alkyl subs­tituent has an all-*trans* conformation [C7—C8—C9—C10 and C8—C9—C10—O1 = −179.8 (3) and −178.7 (3)°, respectively]. In the protonated BIZ mol­ecule, the hy­droxy­alkyl substituent is rotated orthogonally to the benzimidazole fragments [N2—C7—C8—C9 = 103.1 (2)°] and has an *ap*−,−*sc* conformation [C7—C8—C9—C10 and C8—C9—C10—O1 = −179.3 (2) and −62.3 (2)°, respectively].

## Supra­molecular features

**Figure 3:**
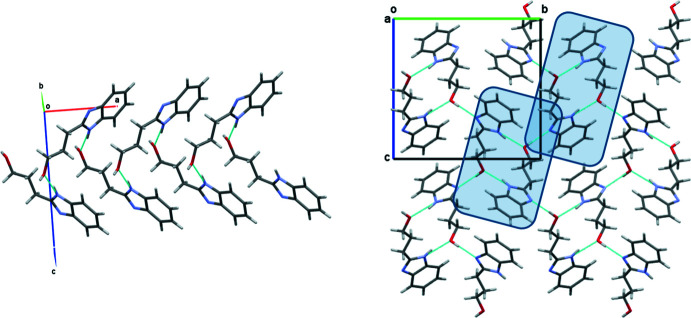
Crystal packing of the neutral mol­ecules in the BIZ structure. Projection in the [100] direction.

In the crystal, BIZ mol­ecules are linked by O—H⋯N and N—H⋯O hydrogen bonds (Table 1 ▸). The zigzag chains formed by the N–H⋯O hydrogen bonds propagate in the [100] direction (Fig. 3 ▸, on the left). These chains are connected by O—H⋯N hydrogen bonds in the [010] and [001] directions (Fig. 3 ▸, on the right; the chains are highlighted in blue). In addition, weak C3—H⋯C3 (π) inter­actions (Table 1 ▸) are observed between the BIZ mol­ecules.

**Figure 4:**
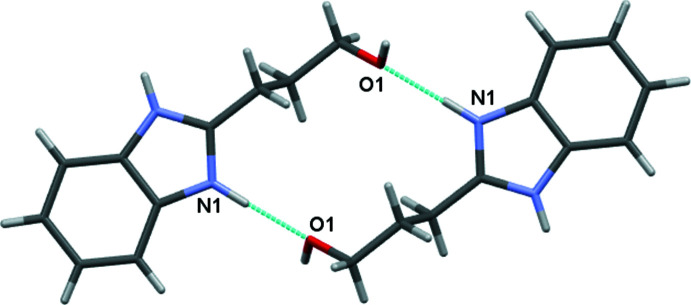
Hydrogen-bonded centrosymmetric dimer of the cations in the nitrate salt. Hydrogen bonds are shown by the cyan lines.

**Figure 5:**
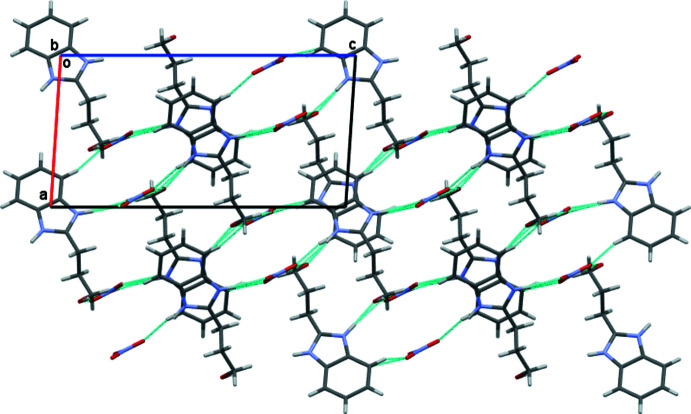
Crystal packing of the 2-(3-hy­droxy­prop­yl)benzimidazole nitrate salt in the BIZHNO3 structure. Projection in the [010] direction. Hydrogen bonds are shown by cyan lines.

In the crystal of the nitrate salt, the protonated BIZ mol­ecules are connected by N—H⋯O hydrogen bonds (Table 2 ▸), forming centrosymmetric dimers (Fig. 4 ▸). These dimers are linked by the bridging nitrate anions in the [001] direction *via* N—H⋯O, O—H⋯O and C—H⋯O hydrogen bonds (Fig. 5 ▸). Stacking inter­actions of the head-to-tail type between the imidazole rings of BIZH^+^ mol­ecules are observed in the [010] direction, the distance between π-systems being 3.502 (2) Å.

## Hirshfeld surface analysis

**Figure 6:**
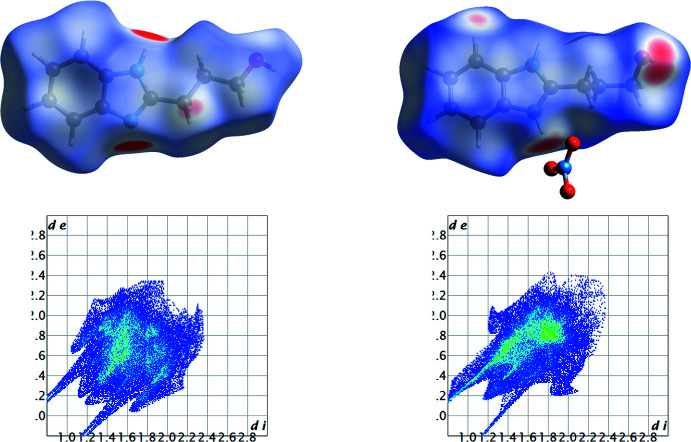
Hirshfeld surfaces mapped over *d*_norm_ (top) and two-dimensional fingerprint plots (bottom) of the neutral 2-(3-hy­droxy­prop­yl)benzimidazole mol­ecule and its protonated cation in the structures of BIZ and BIZHNO3.

Hirshfeld surface analysis (Turner *et al.*, 2017 ▸) is one of the modern methods allowing inter­molecular inter­actions to be studied in a more analytical way. This method appears to be effective for comparing the capability of the neutral BIZ mol­ecule and its protonated form to participate in inter­molecular inter­actions of different types. The Hirshfeld surfaces were calculated for the BIZ and BIZH^+^ mol­ecules using a standard high surface resolution, mapped over *d*_norm_ (Fig. 6 ▸). Bright-red spots are observed for all the donors and acceptors of strong hydrogen bonds in the two structures under study, indicating their participation in inter­molecular inter­actions. It should be noted that the bright-red spot on the N1 atom in the BIZ mol­ecule indicates its capability to be protonated or participate in complexation with a metal.

**Figure 7:**
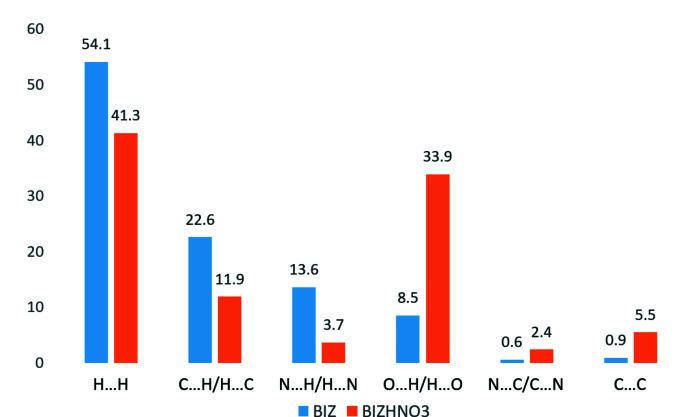
Relative contributions of the strongest inter­molecular inter­actions (in %) to the total Hirshfeld surface of the neutral mol­ecule and its cation in the structures of BIZ and BIZHNO3.

The two-dimensional fingerprint plots constructed for the BIZ and BIZH^+^ mol­ecules show that the hydrogen bonds are stronger in the structure of the nitrate salt (see the sharp spikes in Fig. 6 ▸). To compare inter­molecular inter­actions of different types in the structures under study, we have analysed their contributions to the total Hirshfeld surfaces (Fig. 7 ▸). As can be seen from the histogram, the protonation of the BIZ mol­ecule and presence of the nitrate anion results in a significant increase of the contribution of O⋯H/H⋯O inter­actions associated with *X*—H⋯O hydrogen bonds. In addition, the contributions of N⋯C/C⋯N and C⋯C inter­actions indicate that stacking between imidazole rings also increases in the BIZHNO3 structure (Fig. 7 ▸). A significant decrease in the contribution of N⋯H/H⋯N inter­actions (*X*—H⋯N bonding) in the BIZHNO3 structure can be explained by the protonation of the N1 atom, which participates as proton acceptor of hydrogen bonds in the BIZ structure. The different contributions of C⋯H/H⋯C inter­actions associated with *X*—H⋯C (π) hydrogen bonds coincide with the presence of a C—H⋯C(π) hydrogen bond in the BIZ structure (Table 1 ▸) and the absence of similar inter­actions in the BIZHNO3 structure (Table 2 ▸). The nitrate anions act as bridging moieties in the BIZHNO3 structure, which results in an increase in the distances between BIZH^+^ mol­ecules. This fact can explain the decrease in the contribution of H⋯H inter­actions in the BIZHNO3 structure (Fig. 7 ▸).

## Database survey

A search of the Cambridge Structural Database (CSD, version 5.42, update of November 2020; Groom *et al.*, 2016 ▸) revealed three structures containing the BIZ mol­ecule [refcodes FIYXAN and FIYXER (Elmali *et al.*, 2005 ▸) and RIYNUL (Zhao *et al.*, 2019 ▸)]. Two of these structures (FIYXAN and FIYXER) contain protonated BIZ mol­ecules, which form salts with PtCl_4_^2−^ or PtCl_6_^2−^ anions. In the RIYNUL structure, the BIZ mol­ecule forms a coordination bond with the Cd atom.

In addition, three structures with a close analogue of the BIZ mol­ecule containing a carb­oxy­lic group instead of a hydroxyl group were found in the CSD [refcodes JOQROZ (Fu *et al.*, 2016 ▸), NOVCEI (Liu *et al.*, 2015 ▸) and TILGOL (Zeng *et al.*, 2007 ▸)]. In all of these structures, the organic ligand forms an N—*M* ^+^ coordination bond with participation of the N2 atom of the imidazole ring.

## Synthesis and crystallization

All chemicals were obtained from commercial sources and used directly without further purification. 1,2-Phenyl­enedi­amine (2.16 g, 0.02 mol) was dissolved in hydro­chloric acid (25 mL, 4 *M*) at 373 K, and γ-hy­droxy­butyric acid (2.82 g, 0.02 mol) was added to the solution. The mixture was heated with reflux for 6 h at 398 K. After cooling to room temperature, the mixture was neutralized using NaOH (pH 7–9). The product was dissolved in aqueous ethanol and treated with activated carbon for purification. The 2-hy­droxy­propyl­benzimidazole precipitate was filtered off and dried in air. Pale-beige single crystals of the title compound suitable for X-ray diffraction analysis were recrystallized from ethanol solution by slow evaporation, yield 80%, m.p. 437 K.

**Synthesis of the [BIZH^+^]NO_3_^−^ salt:**

A weighed portion of copper nitrate (3 × 10 ^−3^ mol) was dissolved in a minimum amount of water and mixed with an alcoholic saturated solution of the ligand (6 × 10 ^−3^ mol) while heating in a water bath. The solution turned green. The solution was then acidified with nitric acid to pH 5 to prevent the precipitation of hydroxides. The reaction was carried out for 40 minutes while heating in a water bath, after which the reaction mixture was allowed to crystallize. After three days, the precipitated light-yellow crystals were separated, washed with ethanol, and dried in air. The product yield was 62%, m.p. 371–373 K. 
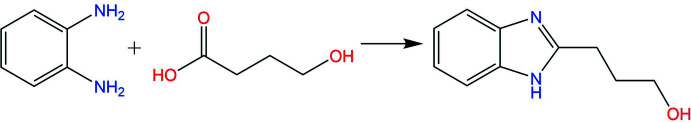


## Refinement

Crystal data, data collection and structure refinement details are summarized in Table 3 ▸. All hydrogen atoms were located in difference-Fourier maps. All of the hydrogen atoms in the BIZ structure and H atoms participating in strong hydrogen bonds in the BIZHNO3 structure were refined using an isotropic approximation. Other hydrogen atoms in the BIZHNO3 structure were refined as riding with C*sp* ^2^—H = 0.97 Å, *U*_iso_(H) = 1.2*U*_eq_(C) for the methyl­ene fragments or Car—H = 0.93 Å, *U*_iso_(H) = 1.2*U*_eq_(C) for the aromatic rings.

## Supplementary Material

Crystal structure: contains datablock(s) BIZ, BIZHNO3. DOI: 10.1107/S2056989022000585/dx2040sup1.cif

Structure factors: contains datablock(s) BIZ. DOI: 10.1107/S2056989022000585/dx2040BIZsup2.hkl

Structure factors: contains datablock(s) BIZHNO3. DOI: 10.1107/S2056989022000585/dx2040BIZHNO3sup3.hkl

Supporting information file. DOI: 10.1107/S2056989022000585/dx2040BIZsup4.cml

Supporting information file. DOI: 10.1107/S2056989022000585/dx2040BIZHNO3sup5.cml

CCDC references: 2142658, 2142657

Additional supporting information:  crystallographic information; 3D view; checkCIF report

## Figures and Tables

**Table 1:** Hydrogen-bond geometry (Å, °) for BIZ[Chem scheme1]

*D*—H⋯*A*	*D*—H	H⋯*A*	*D*⋯*A*	*D*—H⋯*A*
N2—H2*N*⋯O1^i^	0.84 (3)	1.95 (3)	2.772 (3)	165 (3)
O1—H1⋯N1^ii^	0.85 (4)	1.90 (4)	2.741 (3)	169 (4)
C3—H3⋯C3^iii^	0.97 (4)	2.87 (4)	3.770 (4)	154 (3)

Symmetry codes: (i) x+{\script{1\over 2}}, -y+{\script{1\over 2}}, -z+1; (ii) -x+{\script{1\over 2}}, -y+1, z+{\script{1\over 2}}; (iii) x+{\script{1\over 2}}, -y+{\script{1\over 2}}, -z.

**Table 2:** Hydrogen-bond geometry (Å, °) for BIZHNO3

*D*—H⋯*A*	*D*—H	H⋯*A*	*D*⋯*A*	*D*—H⋯*A*
N2—H2*N*⋯O2	0.86 (2)	1.90 (2)	2.755 (2)	173 (2)
N1—H1*N*⋯O1^i^	0.91 (2)	1.78 (2)	2.696 (2)	177 (2)
O1—H1*O*⋯O2^ii^	0.90 (3)	2.06 (3)	2.866 (2)	149 (3)
O1—H1*O*⋯O4^ii^	0.90 (3)	2.14 (3)	2.951 (3)	150 (3)
C5—H5⋯O4^iii^	0.93	2.43	3.251 (3)	147

Symmetry codes: (i) -x+2, -y+1, -z+1; (ii) -x+{\script{3\over 2}}, y+{\script{1\over 2}}, -z+{\script{3\over 2}}; (iii) -x+{\script{1\over 2}}, y-{\script{1\over 2}}, -z+{\script{3\over 2}}.

**Table 3:** Experimental details

	BIZ	BIZHNO3
Crystal data		
Chemical formula	C_10_H_12_N_2_O	C_10_H_13_N_2_O^+^·NO_3_^−^
*M*_r_	176.22	239.23
Crystal system, space group	Orthorhombic, *P*2_1_2_1_2_1_	Monoclinic, *P*2_1_/*n*
Temperature (K)	293	293
*a*, *b*, *c* (Å)	5.852 (2), 12.437 (3), 12.444 (3)	8.5100 (3), 8.2525 (4), 16.5130 (7)
α, β, γ (°)	90, 90, 90	90, 93.760 (4), 90
*V* (Å^3^)	905.7 (4)	1157.19 (9)
*Z*	4	4
Radiation type	Cu *K*α	Cu *K*α
μ (mm^−1^)	0.69	0.91
Crystal size (mm)	0.12 × 0.10 × 0.08	0.12 × 0.10 × 0.08

Data collection		
Diffractometer	Oxford Diffraction Xcallibur, Ruby	Oxford Diffraction Xcalibur, Ruby
Absorption correction	Multi-scan (*CrysAlis PRO*; Oxford Diffraction, 2009 ▸)	Multi-scan (*CrysAlis PRO*; Oxford Diffraction, 2009 ▸)
*T*_min_, *T*_max_	0.958, 1.000	0.739, 1.000
No. of measured, independent and observed [*I* > 2σ(*I*)] reflections	3945, 1371, 1191	4122, 2345, 1644
*R*_int_	0.029	0.021
θ_max_ (°)	62.0	75.8
(sin θ/λ)_max_ (Å^−1^)	0.573	0.629

Refinement		
*R*[*F* ^2^ > 2σ(*F* ^2^)], *wR*(*F* ^2^), *S*	0.039, 0.091, 1.06	0.044, 0.128, 1.04
No. of reflections	1371	2345
No. of parameters	166	167
H-atom treatment	All H-atom parameters refined	H atoms treated by a mixture of independent and constrained refinement
Δρ_max_, Δρ_min_ (e Å^−3^)	0.11, −0.19	0.16, −0.15
Absolute structure	Flack *x* determined using 428 quotients [(*I* ^+^)−(*I* ^−^)]/[(*I* ^+^)+(*I* ^−^)] (Parsons *et al.*, 2013 ▸)	–
Absolute structure parameter	0.1 (3)	–

Computer programs: *CrysAlis PRO* (Oxford Diffraction, 2009 ▸), *SHELXS97* (Sheldrick, 2008 ▸), *SHELXL2016/6* (Sheldrick, 2015 ▸) and *OLEX2* (Dolomanov *et al.*, 2009 ▸).

## supplementary crystallographic information

### 2-(3-Hydroxypropyl)-1H-benzimidazole (BIZ) Crystal data

**Table:**

C_10_H_12_N_2_O	*D*_x_ = 1.292 Mg m^−^^3^
*M**_r_* = 176.22	Cu *K*α radiation, λ = 1.54178 Å
Orthorhombic, *P*2_1_2_1_2_1_	Cell parameters from 1839 reflections
*a* = 5.852 (2) Å	θ = 5.2–32.4°
*b* = 12.437 (3) Å	µ = 0.69 mm^−^^1^
*c* = 12.444 (3) Å	*T* = 293 K
*V* = 905.7 (4) Å^3^	Block, colorless
*Z* = 4	0.12 × 0.10 × 0.08 mm
*F*(000) = 376	

### 2-(3-Hydroxypropyl)-1H-benzimidazole (BIZ) Data collection

**Table:**

Oxford Diffraction Xcallibur, Ruby diffractometer	1371 independent reflections
Radiation source: Enhance (Cu) X-ray Source	1191 reflections with *I* > 2σ(*I*)
Detector resolution: 10.2576 pixels mm^-1^	*R*_int_ = 0.029
ω scans	θ_max_ = 62.0°, θ_min_ = 5.0°
Absorption correction: multi-scan (CrysAlisPro; Oxford Diffraction, 2009)	*h* = −6→6
*T*_min_ = 0.958, *T*_max_ = 1.000	*k* = −13→11
3945 measured reflections	*l* = −12→14

### 2-(3-Hydroxypropyl)-1H-benzimidazole (BIZ) Refinement

**Table:**

Refinement on *F*^2^	Secondary atom site location: difference Fourier map
Least-squares matrix: full	Hydrogen site location: difference Fourier map
*R*[*F*^2^ > 2σ(*F*^2^)] = 0.039	All H-atom parameters refined
*wR*(*F*^2^) = 0.091	*w* = 1/[σ^2^(*F*_o_^2^) + (0.0573*P*)^2^] where *P* = (*F*_o_^2^ + 2*F*_c_^2^)/3
*S* = 1.06	(Δ/σ)_max_ < 0.001
1371 reflections	Δρ_max_ = 0.11 e Å^−^^3^
166 parameters	Δρ_min_ = −0.19 e Å^−^^3^
0 restraints	Absolute structure: Flack *x* determined using 428 quotients [(*I*^+^)-(*I*^-^)]/[(*I*^+^)+(*I*^-^)] (Parsons et al., 2013)
Primary atom site location: difference Fourier map	Absolute structure parameter: 0.1 (3)

### 2-(3-Hydroxypropyl)-1H-benzimidazole (BIZ) Special details

**Table:**

Geometry. All esds (except the esd in the dihedral angle between two l.s. planes) are estimated using the full covariance matrix. The cell esds are taken into account individually in the estimation of esds in distances, angles and torsion angles; correlations between esds in cell parameters are only used when they are defined by crystal symmetry. An approximate (isotropic) treatment of cell esds is used for estimating esds involving l.s. planes.

### 2-(3-Hydroxypropyl)-1H-benzimidazole (BIZ) Fractional atomic coordinates and isotropic or equivalent isotropic displacement parameters (Å^2^)

**Table:**

	*x*	*y*	*z*	*U*_iso_*/*U*_eq_	
O1	−0.0261 (5)	0.38855 (16)	0.5824 (2)	0.0635 (7)	
H1	−0.080 (6)	0.439 (3)	0.621 (3)	0.083 (14)*	
N1	0.6471 (4)	0.43719 (19)	0.2047 (2)	0.0470 (6)	
N2	0.6271 (4)	0.2901 (2)	0.3056 (2)	0.0457 (7)	
H2N	0.587 (5)	0.243 (2)	0.350 (3)	0.045 (9)*	
C1	0.8204 (5)	0.3668 (2)	0.1742 (2)	0.0454 (7)	
C2	0.9878 (6)	0.3766 (3)	0.0962 (3)	0.0559 (9)	
H2	0.994 (6)	0.444 (3)	0.051 (3)	0.068 (10)*	
C3	1.1430 (6)	0.2942 (3)	0.0847 (3)	0.0631 (9)	
H3	1.268 (7)	0.297 (3)	0.034 (3)	0.076 (11)*	
C4	1.1335 (6)	0.2037 (3)	0.1499 (3)	0.0616 (10)	
H4	1.232 (6)	0.148 (3)	0.144 (3)	0.081 (12)*	
C5	0.9683 (6)	0.1915 (3)	0.2277 (3)	0.0555 (9)	
H5	0.961 (6)	0.128 (3)	0.274 (3)	0.075 (11)*	
C6	0.8096 (5)	0.2745 (2)	0.2377 (2)	0.0446 (7)	
C7	0.5365 (5)	0.3875 (2)	0.2830 (2)	0.0427 (7)	
C8	0.3317 (6)	0.4331 (3)	0.3370 (3)	0.0505 (8)	
H8A	0.369 (6)	0.511 (3)	0.353 (3)	0.076 (11)*	
H8B	0.206 (6)	0.431 (3)	0.286 (3)	0.059 (9)*	
C9	0.2555 (6)	0.3800 (3)	0.4403 (3)	0.0485 (8)	
H9A	0.376 (6)	0.382 (3)	0.493 (3)	0.055 (9)*	
H9B	0.220 (5)	0.307 (3)	0.428 (3)	0.049 (8)*	
C10	0.0467 (6)	0.4349 (3)	0.4844 (3)	0.0507 (8)	
H10A	0.073 (5)	0.512 (3)	0.495 (3)	0.051 (8)*	
H10B	−0.086 (6)	0.432 (3)	0.432 (3)	0.069 (10)*	

### 2-(3-Hydroxypropyl)-1H-benzimidazole (BIZ) Atomic displacement parameters (Å^2^)

**Table:**

	*U*^11^	*U*^22^	*U*^33^	*U*^12^	*U*^13^	*U*^23^
O1	0.0956 (18)	0.0401 (12)	0.0547 (14)	0.0140 (12)	0.0202 (13)	0.0041 (11)
N1	0.0527 (15)	0.0425 (12)	0.0457 (15)	−0.0018 (11)	−0.0032 (13)	0.0034 (11)
N2	0.0518 (15)	0.0398 (13)	0.0454 (16)	−0.0046 (11)	−0.0032 (12)	0.0091 (12)
C1	0.0476 (17)	0.0427 (15)	0.0459 (18)	−0.0053 (14)	−0.0035 (14)	−0.0040 (13)
C2	0.064 (2)	0.0517 (18)	0.052 (2)	−0.0131 (17)	0.0045 (16)	−0.0032 (16)
C3	0.060 (2)	0.066 (2)	0.064 (2)	−0.0104 (18)	0.0101 (19)	−0.016 (2)
C4	0.055 (2)	0.057 (2)	0.073 (3)	0.0039 (17)	−0.0013 (18)	−0.0160 (19)
C5	0.059 (2)	0.0442 (17)	0.063 (2)	0.0022 (15)	−0.0082 (18)	−0.0027 (17)
C6	0.0465 (16)	0.0425 (15)	0.0448 (18)	−0.0059 (13)	−0.0059 (14)	−0.0025 (13)
C7	0.0468 (16)	0.0404 (14)	0.0409 (16)	−0.0043 (13)	−0.0073 (14)	−0.0007 (13)
C8	0.0551 (18)	0.0509 (17)	0.0455 (18)	0.0022 (16)	−0.0049 (16)	0.0033 (14)
C9	0.058 (2)	0.0418 (18)	0.046 (2)	0.0018 (14)	−0.0023 (15)	−0.0023 (15)
C10	0.0614 (19)	0.0441 (17)	0.0467 (19)	0.0030 (16)	−0.0008 (17)	0.0035 (15)

### 2-(3-Hydroxypropyl)-1H-benzimidazole (BIZ) Geometric parameters (Å, º)

**Table:**

O1—C10	1.414 (4)	C4—C5	1.376 (5)
O1—H1	0.85 (4)	C4—H4	0.91 (4)
N1—C7	1.322 (4)	C5—C6	1.394 (4)
N1—C1	1.393 (4)	C5—H5	0.98 (4)
N2—C7	1.352 (4)	C7—C8	1.486 (5)
N2—C6	1.376 (4)	C8—C9	1.513 (4)
N2—H2N	0.84 (3)	C8—H8A	1.02 (4)
C1—C2	1.385 (4)	C8—H8B	0.97 (3)
C1—C6	1.395 (4)	C9—C10	1.503 (5)
C2—C3	1.377 (5)	C9—H9A	0.96 (4)
C2—H2	1.01 (4)	C9—H9B	0.94 (3)
C3—C4	1.389 (5)	C10—H10A	0.98 (3)
C3—H3	0.97 (4)	C10—H10B	1.01 (4)
			
C10—O1—H1	107 (3)	C5—C6—C1	121.9 (3)
C7—N1—C1	105.3 (2)	N1—C7—N2	112.3 (3)
C7—N2—C6	107.6 (3)	N1—C7—C8	123.4 (3)
C7—N2—H2N	131 (2)	N2—C7—C8	124.3 (3)
C6—N2—H2N	121 (2)	C7—C8—C9	117.1 (3)
C2—C1—N1	130.6 (3)	C7—C8—H8A	106 (2)
C2—C1—C6	120.1 (3)	C9—C8—H8A	109 (2)
N1—C1—C6	109.3 (3)	C7—C8—H8B	107 (2)
C3—C2—C1	118.3 (3)	C9—C8—H8B	109 (2)
C3—C2—H2	123 (2)	H8A—C8—H8B	109 (3)
C1—C2—H2	119 (2)	C10—C9—C8	110.6 (3)
C2—C3—C4	121.1 (4)	C10—C9—H9A	109.7 (19)
C2—C3—H3	123 (2)	C8—C9—H9A	111 (2)
C4—C3—H3	116 (2)	C10—C9—H9B	108.7 (19)
C5—C4—C3	121.9 (4)	C8—C9—H9B	110.1 (19)
C5—C4—H4	115 (2)	H9A—C9—H9B	107 (3)
C3—C4—H4	123 (2)	O1—C10—C9	112.0 (3)
C4—C5—C6	116.7 (3)	O1—C10—H10A	109.1 (19)
C4—C5—H5	122 (2)	C9—C10—H10A	111.5 (19)
C6—C5—H5	121 (2)	O1—C10—H10B	108 (2)
N2—C6—C5	132.6 (3)	C9—C10—H10B	112 (2)
N2—C6—C1	105.5 (3)	H10A—C10—H10B	104 (3)
			
C7—N1—C1—C2	179.9 (3)	N1—C1—C6—N2	0.5 (3)
C7—N1—C1—C6	−0.5 (3)	C2—C1—C6—C5	2.1 (4)
N1—C1—C2—C3	178.5 (3)	N1—C1—C6—C5	−177.5 (3)
C6—C1—C2—C3	−1.0 (4)	C1—N1—C7—N2	0.3 (3)
C1—C2—C3—C4	−0.4 (5)	C1—N1—C7—C8	−177.9 (3)
C2—C3—C4—C5	0.8 (5)	C6—N2—C7—N1	0.0 (3)
C3—C4—C5—C6	0.2 (5)	C6—N2—C7—C8	178.2 (3)
C7—N2—C6—C5	177.5 (3)	N1—C7—C8—C9	−166.7 (3)
C7—N2—C6—C1	−0.3 (3)	N2—C7—C8—C9	15.3 (4)
C4—C5—C6—N2	−179.1 (3)	C7—C8—C9—C10	−179.8 (3)
C4—C5—C6—C1	−1.7 (5)	C8—C9—C10—O1	−178.7 (3)
C2—C1—C6—N2	−179.9 (3)		

### 2-(3-Hydroxypropyl)-1H-benzimidazole (BIZ) Hydrogen-bond geometry (Å, º)

**Table:**

*D*—H···*A*	*D*—H	H···*A*	*D*···*A*	*D*—H···*A*
N2—H2*N*···O1^i^	0.84 (3)	1.95 (3)	2.772 (3)	165 (3)
O1—H1···N1^ii^	0.85 (4)	1.90 (4)	2.741 (3)	169 (4)
C3—H3···C3^iii^	0.97 (4)	2.87 (4)	3.770 (4)	154 (3)

Symmetry codes: (i) *x*+1/2, −*y*+1/2, −*z*+1; (ii) −*x*+1/2, −*y*+1, *z*+1/2; (iii) *x*+1/2, −*y*+1/2, −*z*.

### 2-(3-Hydroxypropyl)-1H-benzimidazol-3-ium nitrate (BIZHNO3) Crystal data

**Table:**

C_10_H_13_N_2_O^+^·NO_3_^−^	*F*(000) = 504
*M**_r_* = 239.23	*D*_x_ = 1.373 Mg m^−^^3^
Monoclinic, *P*2_1_/*n*	Cu *K*α radiation, λ = 1.54184 Å
*a* = 8.5100 (3) Å	Cell parameters from 1670 reflections
*b* = 8.2525 (4) Å	θ = 5.2–75.5°
*c* = 16.5130 (7) Å	µ = 0.91 mm^−^^1^
β = 93.760 (4)°	*T* = 293 K
*V* = 1157.19 (9) Å^3^	Block, colorless
*Z* = 4	0.12 × 0.10 × 0.08 mm

### 2-(3-Hydroxypropyl)-1H-benzimidazol-3-ium nitrate (BIZHNO3) Data collection

**Table:**

Oxford Diffraction Xcalibur, Ruby diffractometer	2345 independent reflections
Radiation source: Enhance (Cu) X-ray Source	1644 reflections with *I* > 2σ(*I*)
Detector resolution: 10.2576 pixels mm^-1^	*R*_int_ = 0.021
ω scans	θ_max_ = 75.8°, θ_min_ = 5.4°
Absorption correction: multi-scan (CrysAlisPro; Oxford Diffraction, 2009)	*h* = −9→10
*T*_min_ = 0.739, *T*_max_ = 1.000	*k* = −10→8
4122 measured reflections	*l* = −20→18

### 2-(3-Hydroxypropyl)-1H-benzimidazol-3-ium nitrate (BIZHNO3) Refinement

**Table:**

Refinement on *F*^2^	Secondary atom site location: difference Fourier map
Least-squares matrix: full	Hydrogen site location: mixed
*R*[*F*^2^ > 2σ(*F*^2^)] = 0.044	H atoms treated by a mixture of independent and constrained refinement
*wR*(*F*^2^) = 0.128	*w* = 1/[σ^2^(*F*_o_^2^) + (0.0715*P*)^2^ + 0.0026*P*] where *P* = (*F*_o_^2^ + 2*F*_c_^2^)/3
*S* = 1.04	(Δ/σ)_max_ < 0.001
2345 reflections	Δρ_max_ = 0.16 e Å^−^^3^
167 parameters	Δρ_min_ = −0.15 e Å^−^^3^
0 restraints	Extinction correction: SHELXL2016/6 (Sheldrick 2015), Fc^*^=kFc[1+0.001xFc^2^λ^3^/sin(2θ)]^-1/4^
Primary atom site location: difference Fourier map	Extinction coefficient: 0.0075 (11)

### 2-(3-Hydroxypropyl)-1H-benzimidazol-3-ium nitrate (BIZHNO3) Special details

**Table:**

Geometry. All esds (except the esd in the dihedral angle between two l.s. planes) are estimated using the full covariance matrix. The cell esds are taken into account individually in the estimation of esds in distances, angles and torsion angles; correlations between esds in cell parameters are only used when they are defined by crystal symmetry. An approximate (isotropic) treatment of cell esds is used for estimating esds involving l.s. planes.

### 2-(3-Hydroxypropyl)-1H-benzimidazol-3-ium nitrate (BIZHNO3) Fractional atomic coordinates and isotropic or equivalent isotropic displacement parameters (Å^2^)

**Table:**

	*x*	*y*	*z*	*U*_iso_*/*U*_eq_	
O1	1.12121 (17)	0.57042 (19)	0.61912 (9)	0.0691 (5)	
H1O	1.102 (4)	0.662 (3)	0.6461 (18)	0.112 (10)*	
O2	0.4689 (2)	0.27088 (18)	0.74998 (8)	0.0732 (5)	
O3	0.4922 (3)	0.5257 (2)	0.74234 (10)	0.1045 (7)	
O4	0.3822 (2)	0.4195 (2)	0.84232 (10)	0.1011 (6)	
N1	0.65129 (19)	0.34198 (19)	0.47832 (10)	0.0519 (4)	
H1N	0.726 (3)	0.374 (3)	0.4442 (14)	0.075 (7)*	
N2	0.54243 (17)	0.29223 (18)	0.59032 (9)	0.0477 (4)	
H2N	0.527 (3)	0.290 (3)	0.6412 (13)	0.065 (6)*	
C1	0.5164 (2)	0.2529 (2)	0.45731 (11)	0.0464 (4)	
C2	0.4513 (3)	0.1965 (2)	0.38345 (12)	0.0591 (5)	
H2	0.498209	0.216950	0.335175	0.071*	
N3	0.4465 (2)	0.4091 (2)	0.77860 (10)	0.0616 (4)	
C3	0.3151 (3)	0.1095 (2)	0.38500 (13)	0.0663 (6)	
H3	0.268665	0.069383	0.336521	0.080*	
C4	0.2433 (2)	0.0789 (2)	0.45704 (14)	0.0641 (6)	
H4	0.149566	0.020639	0.455243	0.077*	
C5	0.3081 (2)	0.1330 (2)	0.53068 (12)	0.0541 (5)	
H5	0.261444	0.111399	0.578915	0.065*	
C6	0.44674 (19)	0.22143 (19)	0.52923 (10)	0.0444 (4)	
C7	0.6645 (2)	0.3629 (2)	0.55826 (11)	0.0486 (4)	
C8	0.7967 (2)	0.4460 (2)	0.60431 (12)	0.0576 (5)	
H8A	0.765574	0.471662	0.658317	0.069*	
H8B	0.819160	0.547104	0.577420	0.069*	
C9	0.9449 (2)	0.3426 (2)	0.61118 (13)	0.0567 (5)	
H9A	0.921976	0.240986	0.637422	0.068*	
H9B	0.976665	0.318146	0.557167	0.068*	
C10	1.0784 (2)	0.4255 (3)	0.65864 (12)	0.0618 (5)	
H10A	1.047114	0.450854	0.712636	0.074*	
H10B	1.168414	0.353286	0.664051	0.074*	

### 2-(3-Hydroxypropyl)-1H-benzimidazol-3-ium nitrate (BIZHNO3) Atomic displacement parameters (Å^2^)

**Table:**

	*U*^11^	*U*^22^	*U*^33^	*U*^12^	*U*^13^	*U*^23^
O1	0.0677 (10)	0.0726 (10)	0.0693 (9)	−0.0224 (8)	0.0217 (7)	−0.0162 (8)
O2	0.1068 (13)	0.0561 (8)	0.0580 (8)	0.0013 (8)	0.0154 (8)	−0.0021 (7)
O3	0.1610 (19)	0.0591 (10)	0.0956 (13)	−0.0156 (11)	0.0260 (13)	0.0054 (9)
O4	0.1175 (15)	0.1153 (15)	0.0749 (11)	0.0069 (12)	0.0408 (10)	−0.0192 (10)
N1	0.0507 (9)	0.0494 (8)	0.0569 (9)	−0.0031 (7)	0.0136 (7)	0.0017 (7)
N2	0.0479 (8)	0.0489 (8)	0.0468 (8)	−0.0003 (6)	0.0071 (7)	−0.0006 (7)
C1	0.0469 (9)	0.0403 (8)	0.0521 (9)	0.0048 (7)	0.0040 (7)	0.0032 (7)
C2	0.0714 (13)	0.0556 (11)	0.0498 (10)	0.0078 (10)	−0.0003 (9)	0.0008 (9)
N3	0.0658 (11)	0.0633 (10)	0.0556 (10)	0.0006 (8)	0.0026 (8)	−0.0041 (8)
C3	0.0695 (14)	0.0583 (12)	0.0681 (13)	0.0051 (10)	−0.0170 (11)	−0.0093 (10)
C4	0.0478 (11)	0.0521 (11)	0.0911 (16)	−0.0028 (9)	−0.0055 (10)	−0.0061 (10)
C5	0.0473 (10)	0.0466 (10)	0.0691 (12)	−0.0012 (8)	0.0096 (9)	0.0020 (9)
C6	0.0433 (9)	0.0393 (8)	0.0509 (9)	0.0033 (7)	0.0041 (7)	0.0011 (7)
C7	0.0453 (9)	0.0427 (9)	0.0582 (10)	0.0022 (7)	0.0057 (8)	−0.0029 (8)
C8	0.0489 (11)	0.0524 (10)	0.0715 (13)	−0.0021 (8)	0.0041 (9)	−0.0132 (9)
C9	0.0548 (11)	0.0457 (9)	0.0685 (11)	0.0003 (8)	−0.0027 (9)	−0.0031 (9)
C10	0.0558 (11)	0.0662 (12)	0.0622 (12)	0.0003 (10)	−0.0053 (9)	−0.0056 (10)

### 2-(3-Hydroxypropyl)-1H-benzimidazol-3-ium nitrate (BIZHNO3) Geometric parameters (Å, º)

**Table:**

O1—C10	1.421 (2)	C3—C4	1.396 (3)
O1—H1O	0.90 (3)	C3—H3	0.9300
O2—N3	1.254 (2)	C4—C5	1.376 (3)
O3—N3	1.211 (2)	C4—H4	0.9300
O4—N3	1.220 (2)	C5—C6	1.389 (2)
N1—C7	1.329 (2)	C5—H5	0.9300
N1—C1	1.388 (2)	C7—C8	1.484 (2)
N1—H1N	0.91 (2)	C8—C9	1.521 (3)
N2—C7	1.331 (2)	C8—H8A	0.9700
N2—C6	1.384 (2)	C8—H8B	0.9700
N2—H2N	0.86 (2)	C9—C10	1.502 (3)
C1—C6	1.386 (2)	C9—H9A	0.9700
C1—C2	1.387 (3)	C9—H9B	0.9700
C2—C3	1.365 (3)	C10—H10A	0.9700
C2—H2	0.9300	C10—H10B	0.9700
			
C10—O1—H1O	114.7 (19)	N2—C6—C1	106.33 (15)
C7—N1—C1	109.37 (16)	N2—C6—C5	132.01 (17)
C7—N1—H1N	124.0 (14)	C1—C6—C5	121.66 (17)
C1—N1—H1N	126.4 (14)	N1—C7—N2	108.73 (16)
C7—N2—C6	109.41 (15)	N1—C7—C8	125.51 (17)
C7—N2—H2N	125.0 (15)	N2—C7—C8	125.72 (17)
C6—N2—H2N	125.6 (15)	C7—C8—C9	112.10 (15)
C6—C1—C2	121.46 (17)	C7—C8—H8A	109.2
C6—C1—N1	106.16 (15)	C9—C8—H8A	109.2
C2—C1—N1	132.37 (18)	C7—C8—H8B	109.2
C3—C2—C1	116.80 (19)	C9—C8—H8B	109.2
C3—C2—H2	121.6	H8A—C8—H8B	107.9
C1—C2—H2	121.6	C10—C9—C8	112.27 (16)
O3—N3—O4	123.2 (2)	C10—C9—H9A	109.2
O3—N3—O2	118.34 (18)	C8—C9—H9A	109.2
O4—N3—O2	118.47 (18)	C10—C9—H9B	109.2
C2—C3—C4	122.06 (19)	C8—C9—H9B	109.2
C2—C3—H3	119.0	H9A—C9—H9B	107.9
C4—C3—H3	119.0	O1—C10—C9	110.57 (17)
C5—C4—C3	121.47 (19)	O1—C10—H10A	109.5
C5—C4—H4	119.3	C9—C10—H10A	109.5
C3—C4—H4	119.3	O1—C10—H10B	109.5
C4—C5—C6	116.53 (18)	C9—C10—H10B	109.5
C4—C5—H5	121.7	H10A—C10—H10B	108.1
C6—C5—H5	121.7		
			
C7—N1—C1—C6	0.46 (19)	N1—C1—C6—C5	−179.68 (15)
C7—N1—C1—C2	−178.67 (19)	C4—C5—C6—N2	−179.65 (17)
C6—C1—C2—C3	0.4 (3)	C4—C5—C6—C1	−0.3 (3)
N1—C1—C2—C3	179.40 (19)	C1—N1—C7—N2	−0.6 (2)
C1—C2—C3—C4	0.4 (3)	C1—N1—C7—C8	177.29 (16)
C2—C3—C4—C5	−1.1 (3)	C6—N2—C7—N1	0.50 (19)
C3—C4—C5—C6	1.0 (3)	C6—N2—C7—C8	−177.38 (16)
C7—N2—C6—C1	−0.21 (18)	N1—C7—C8—C9	−74.5 (2)
C7—N2—C6—C5	179.26 (18)	N2—C7—C8—C9	103.1 (2)
C2—C1—C6—N2	179.09 (16)	C7—C8—C9—C10	−179.28 (17)
N1—C1—C6—N2	−0.15 (18)	C8—C9—C10—O1	−62.3 (2)
C2—C1—C6—C5	−0.4 (3)		

### 2-(3-Hydroxypropyl)-1H-benzimidazol-3-ium nitrate (BIZHNO3) Hydrogen-bond geometry (Å, º)

**Table:**

*D*—H···*A*	*D*—H	H···*A*	*D*···*A*	*D*—H···*A*
N2—H2*N*···O2	0.86 (2)	1.90 (2)	2.755 (2)	173 (2)
N1—H1*N*···O1^i^	0.91 (2)	1.78 (2)	2.696 (2)	177 (2)
O1—H1*O*···O2^ii^	0.90 (3)	2.06 (3)	2.866 (2)	149 (3)
O1—H1*O*···O4^ii^	0.90 (3)	2.14 (3)	2.951 (3)	150 (3)
C5—H5···O4^iii^	0.93	2.43	3.251 (3)	147

Symmetry codes: (i) −*x*+2, −*y*+1, −*z*+1; (ii) −*x*+3/2, *y*+1/2, −*z*+3/2; (iii) −*x*+1/2, *y*−1/2, −*z*+3/2.
